# CRISPR/gRNA-directed synergistic activation mediator (SAM) induces specific, persistent and robust reactivation of the HIV-1 latent reservoirs

**DOI:** 10.1038/srep16277

**Published:** 2015-11-05

**Authors:** Yonggang Zhang, Chaoran Yin, Ting Zhang, Fang Li, Wensheng Yang, Rafal Kaminski, Philip Regis Fagan, Raj Putatunda, Won-Bin Young, Kamel Khalili, Wenhui Hu

**Affiliations:** 1 Department of Neuroscience, Center for Neurovirology and The Comprehensive NeuroAIDS Center, Temple University School of Medicine, 3500 N Broad Street, Philadelphia, PA 19140; 2 Department of Radiology, University of Pittsburgh School of Medicine, Pittsburgh, PA, 15219.

## Abstract

Current antiretroviral therapy does not eliminate the integrated and transcriptionally silent HIV-1 provirus in latently infected cells. Recently, a “shock and kill” strategy has been extensively explored to eradicate the HIV-1 latent reservoirs for a permanent cure of AIDS. The therapeutic efficacy of currently used agents remains disappointing because of low efficiency, non-specificity and cellular toxicity. Here we present a novel catalytically-deficient Cas9-synergistic activation mediator (dCas9-SAM) technology to selectively, potently and persistently reactivate the HIV-1 latent reservoirs. By screening 16 MS2-mediated single guide RNAs, we identified long terminal repeat (LTR)-L and O that surround the enhancer region (-165/-145 for L and -92/-112 for O) and induce robust reactivation of HIV-1 provirus in HIV-1 latent TZM-bI epithelial, Jurkat T lymphocytic and CHME5 microglial cells. This compulsory reactivation induced cellular suicide via toxic buildup of viral proteins within HIV-1 latent Jurkat T and CHME5 microglial cells. These results suggest that this highly effective and target-specific dCas9-SAM system can serve as a novel HIV-latency-reversing therapeutic tool for the permanent elimination of HIV-1 latent reservoirs.

HIV-1 infection remains a major public health problem affecting more than 35 million people worldwide and more than 1.2 million people in the United States. Combined antiretroviral therapy (cART) can achieve a “functional cure”, but HIV-1 resurgence in latently infected cells after cART withdrawal is a main obstacle to a permanent cure of HIV-1 infection. Current cART does not eliminate the integrated and transcriptionally silent HIV-1 provirus in latently infected cells. The most current strategy (dubbed “shock (kick) and kill”) is aimed to reactivate latently infected cells and induce their subsequent cell death due to viral cytotoxicity and/or host immune defense. The following concomitant and pulsed antiretroviral treatment after the “shock” treatment will prevent virus spread and block new infection^1^. Several agents or small molecules, in particular, the histone deacetylase (HDAC) inhibitors, have been developed to reactivate the HIV-1 latent reservoirs, some of which are currently tested in clinical trials^2^,^3^,^4^,^5^. However, the reactivation results are not as promising as expected, probably due to insufficient reactivation, non-specific cell targeting, apoptosis resistance and drug toxicity^3^,^4^,^6^. For example, a recent report using a humanized HIV-1 latency mouse model demonstrated that only a combined treatment with three well-established latency-reversing agents including HDAC inhibitor suberoylanilide hydroxamic acid (SAHA), the BET bromodomain protein inhibitor I-BET151, and the immune modulatory anti-CTLA4 antibody, allowed a sufficient level of HIV-1 upregulation in HIV-1 latent cells for the elimination by the broadly neutralizing anti-HIV-1 antibodies^7^. Multiple latency-reversing agents via several signal transduction pathways^8^ would increase the toxicity to HIV-negative cells, similar to the chemotherapy treatment of cancerous cells. Furthermore, repeated administration of these latency-reversing agents is required to maintain a continuous reactivation of the HIV-1 latent reservoirs. Therefore, a better reactivator of latent HIV-1 provirus, which displays targeted cell specificity, high efficiency and no/low cytotoxicity remains to be identified. To achieve HIV-targeted specific reactivation, ZFN and TALEN have been tested by engineering target-specific activators such as VP64^9^,^10^, but the efficiency is relatively low. So far, there have been no reports on catalytically-deficient Cas9 (dCas9)-mediated HIV-1 reactivation, particularly the dCas9-synergistic activation mediator (dCas9-SAM) system^11^, although a recent review discussed its plausibility as an effective HIV-1 therapy^12^. Given that RNA-guided CRISPR/Cas9 technology is simple and inexpensive, we hypothesized that dCas9-mediated reactivation might be a novel practical, specific and personalized remedy for the “shock and kill” strategy to cure HIV/AIDS.

Successful application of CRISPR/Cas9 technology to the mammalian system for genome editing was first reported in early 2013^13^,^14^. Since then, this novel genome editing system has attracted a huge amount of attention in biomedical field, and subsequent examples of this system’s effectiveness have been seen in the fields of animal models, genetic diseases, cancer biology and infectious diseases^12^,^15^,^16^,^17^,^18^. Simultaneously, the use of dCas9 conjugated with a single transcriptional activator or repressor to manipulate cellular gene regulation has been developed^19^,^20^,^21^,^22^. However, this single regulator system has its limitations, such as effectiveness of gene activation/repression and scalability. Thus, recruitment of multiplex transcriptional activators through guide RNA (gRNA) modification and/or dCas9 fusion has been explored^23^,^24^,^25^. Recently, a newer and technologically more advanced dCas9-based SAM system has been developed by engineering the single gRNA (sgRNA) through appending a minimal hairpin aptamer to the tetraloop and stem loop 2 of sgRNA^11^. Such an aptamer is capable of binding to the dimerized MS2 bacteriophage coat proteins. By fusing MS2 proteins with various activators such as p65 and HSF1 transactivation domains, a novel MS2-p65-HSF1 complex guided by target-specific MS2-mediated sgRNA (msgRNA) will enhance the recruitment of transcription factors around the target gene promoter and thus facilitate the potency of dCas9-mediated gene activation by up to 3,000 fold^11^. In this study, we explored the feasibility of this dCas9-SAM technology to activate HIV-1 long terminal repeat (LTR) promoter in HIV-1 latent cells. We identified two msgRNAs that exhibited very robust and sustained reactivation of HIV-1 latent viruses. The target-specific compulsory reactivation leads to suicide of the HIV-1-infected cells. Such a specific and potent reactivation of HIV-1 latent reservoirs may add a newer and more realistic alternative to the “shock and kill” strategy to potentially achieve a permanent cure of HIV/AIDS.

## Results

### Bioinformatic screening of effective sgRNAs that guide dCas9-single activator to the HIV-1 LTR promoter

**Figure 1:**
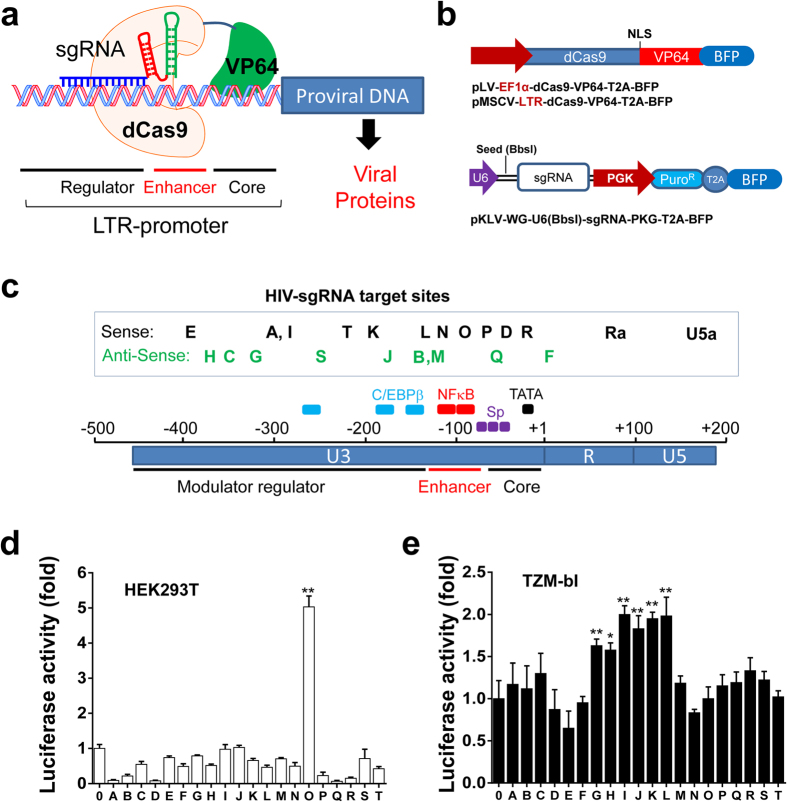
Screening of single guide RNAs (sgRNAs) that guide dCas9-transcriptional activator to the HIV-1 long terminal repeat (LTR) promoter. (**a**) Illustration of sgRNA-guided HIV-1 genome regulation platform for dCas9 fusion with transcriptional activator (VP64). (**b**) Diagram of dCas9-VP64 expressing lentiviral or retroviral vector and sgRNA expressing lentiviral vector (LV). (**c**) Illustration of sgRNA targeting locations within HIV-1 LTR. Some of transcription factors are shown in different colors. (**d**,**e**) Identification of effective sgRNAs with dCas9-VP64 in HEK293T cells transiently coexpressing NL4-3-EcoHIV-*firefly* luciferase (eLuc) reporter (**d**) or TZM-bI cells integrated with LTR-eLuc reporter (**e**). At 2 days after cotransfection, ONE-Glo™ luciferase assay was performed. Data represent mean ± SEM of 4 independent transfections, showing fold changes in luminescent reporter activity relative to corresponding empty sgRNA expression vector (LTR-0). *p < 0.05 and **p < 0.01 indicate statistical significance by ANOVA and FLSD test.

The dCas9 has been widely explored via fusion with transcriptional activators (e.g. VP64, p65) or repressor (e.g. KRAB)^19^,^20^,^21^,^22^ to regulate transcriptional activation or repression of cellular genes through target gene-specific sgRNAs. To provide a similar proof of concept in viral replication/infection regulation (Fig. 1a,b), we designed 20 sgRNA target sites within the HIV-1 promoter (LTR-U3) region using Cas9-sgRNA design tools for best scores of high efficiency and high specificity (Fig. 1c). We cloned these seed sequences into a sgRNA expression lentiviral vector (Fig. 1b). We utilized EcoHIV-*firefly*-luciferase (eLuc) reporter assay for the sensitive, easy screening and the similarity to the HIV NL4.3 virus. We cotransfected one of these sgRNA lentiviral vectors with the dCas9-VP64 lentiviral vector plus a pNL4-3-EcoHIV-eLuc reporter (1:20 to ensure the expression of dCas9-VP64/sgRNA in each reporter-expressing cells) in HEK293T cells, and measured the production of the EcoHIV reporter virus using OneGlo eLuc luminescence assay at 2 days post-transfection. We found that only LTR-O sgRNA induced a 5-fold increase in reporter activation but the majority of these sgRNAs exhibited a marginal change in reporter activities (Fig. 1d). To determine if the dCas9-VP64/LTR-sgRNA system effectively reactivates the latent HIV-1 provirus, we utilized a TZM-bI cell line that contains an integrated LTR-eLuc reporter gene, which is a well-established model to study HIV-1 latency reactivation^26^. Through cotransfection of dCas9-VP64 with indicated sgRNA expressing vectors via lipofectamine 3000 (to help maximize transfection efficiency), we found marginal levels of reactivation (1.5–2 fold) in a few sgRNAs LTR-G to LTR-L (Fig. 1e). These data suggest that most of the selected LTR-sgRNAs with the dCas9-single activator system do not remarkably activate HIV-1 LTR promoter, with LTR-I, J, K, L showing highest, but marginal reactivation of the HIV-1 LTR latent reporter.

### Screening of effective msgRNAs that guide dCas9-SAM to activate HIV-1 LTR promoter

**Figure 2:**
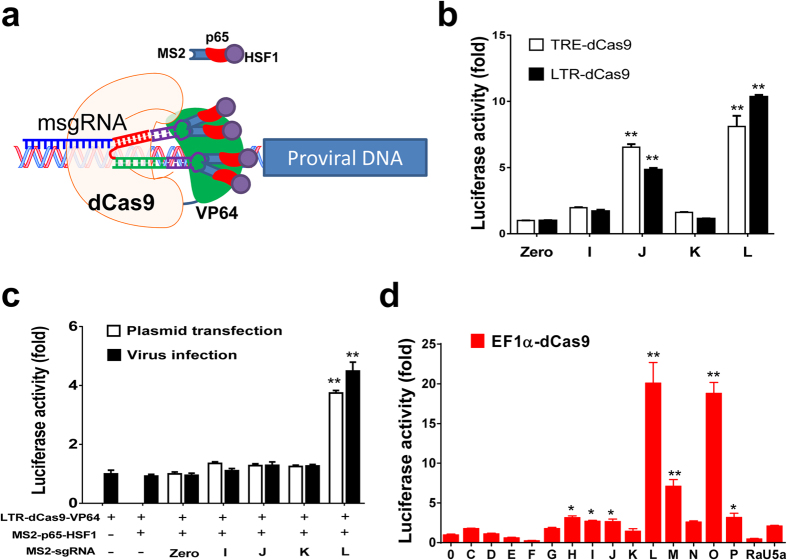
Screening of MS2-mediated sgRNAs (msgRNAs) targeting the HIV-1 long terminal repeat (LTR) in the dCas9-synergistic activation mediator (SAM) system. (**a**) Illustration of SAM-VPH complex derived from dCas9-VP64 (V) and MS2-p65-HSF1 (PH) with msgRNA. (**b**) Similar activation of transfected EcoHIV-eLuc reporter by inducible (TRE) or constitutive (LTR) dCas9-VP64 expression in HEK293T cells. (**c**) Similar reactivation of latent HIV-LTR-eLuc reporter in TZM-bI cells after either transient plasmid transfection by Lipofectamine 3000 or stable infection by lentivirus. Note that MS2-p65-HSF1 alone did not influence the basal activity of LTR-eLuc reporter. The msgRNA-J has no effect due to 2 nucleotide mismatches to the LTR sequence from TZM-bI cells. (**d**) Identification of msgRNA LTR-L and O with best efficiency to activate EcoHIV-eLuc reporter in HEK239T cells with dCas9-SAM system. Data represent mean ± SEM of 4–6 independent transfections or infections, showing fold changes relative to corresponding empty msgRNA expression vector (LTR-0). *p < 0.05 and **p < 0.01 indicate statistical significance by ANOVA and FLSD test.

In cellular genes, a complex of multiple transcriptional activators showed much stronger activation than the single dCas9-transcription factor itself^11^,^27^,^28^. Thus, we applied the newly-developed MS2-mediated SAM system (Fig. 2a), which combined dCas9-VP64 with MS2-p65-HSF1 clustered *trans*-activators, designated dCas9-VPH^11^. We first tested the feasibility of dCas9-VPH in facilitating LTR promoter activation by cloning the seed sequence of the effective sgRNAs LTR-I, J, K, L into the msgRNA-expressing lentiviral vector. This uses the same sgRNA expression cassette incorporated with 2 hairpin aptamers selectively bound to dimerized MS2-fusion proteins at the sgRNA tetraloop and stem loop 2. Such a design allows the recruitment of 4 copies of MS2-fusion protein to a single msgRNA. By cotransfection of pMSCV-LTR-dCas9-VP64-BFP/pLV-MS2-p65-HSF1, and pNL4-3-EcoHIV-eLuc vectors with each of msgRNAs LTR-I, J, K and L in HEK293T cells, we found that LTR-J and L increased the EcoHIV eLuc reporter activity by 6–7 fold compared to the empty msgRNA LTR-Zero control, but msgRNA LTR-I and K showed no noticeable activation of the EcoHIV-reporter (Fig. 2b). This result was further confirmed by inducible expression of dCas9-VP64 to replace the constitutive dCas9-VP64 (Fig. 2b). To determine if dCas9-VPH/msgRNAs induced a better reactivation of HIV-1 latency, we cotransfected or cotransduced HIV-latent TZM-bI cells with dCas9-VPH and indicated msgRNAs (Fig. 2c) and measured the latency-reversing capability. The msgRNA LTR-L expression by Lipofectamine 3000 transfection effectively induced a 3.7-fold reactivation of latent HIV-1 LTR eLuc reporter activity and lentiviral transduction of these components for msgRNA-L in TZM-bI cells caused a 4.5-fold increase in HIV-1 LTR-promoter activity (Fig. 2c). These data suggest that dCas9-VPH exhibits better reactivation than dCas9-VP64 alone in HIV-1 latent reporter, which also relies on gene delivery efficiency. The absence of reactivation by msgRNA LTR-J in TZM-bI cells in contrast to EcoHIV-eLuc reporter in HEK293T cells reflected the fact that one mismatch was found by PCR cloning and Sanger sequencing in the target sequence of integrated LTR in TZM-bI cells^29^.

To further screen for the best msgRNAs with maximal reactivation efficiency within the HIV-1 LTR, we cloned the seed sequences of the sgRNAs as described above and two additional seed sequences targeting the R and U5 regions (Supplemental Table S1), which also contain an enhancer for LTR promoter regulation. We transiently co-transfected them with pLV-EF1α-dCas9-VP64-BFP/pLV-MS2-p65-HSF1 into HEK293T cells in the presence of a pNL4-3-EcoHIV-eLuc reporter vector. We found that 12 of 16 designed msgRNAs increased HIV-eLuc activity but only L and O substantially up-regulated the eLuc reporter expression by up to 20-fold at 2 days after transfection (Fig. 2d). Taken together, these sets of data suggest that the MS2-mediated dCas9-SAM system dramatically improves the efficiency of promoting HIV-1 transcription, and msgRNAs LTR-L and O are the most effective among the tested msgRNAs.

### Robust reactivation by LTR-L/O msgRNAs in HIV-1 latent TZM-bI cells

**Figure 3:**
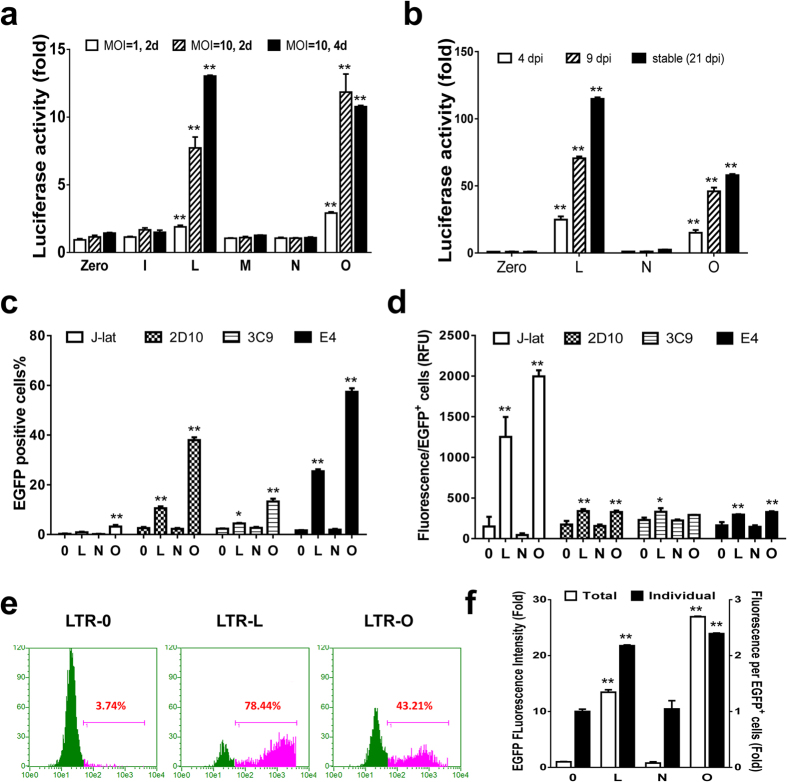
Robust reactivation of HIV-1 LTR promoter by lentivirus-mediated dCas9-SAM in HIV-1 latent cell lines. (**a,b**) TZM-bI cells were infected with dCas9-VPH and indicated msgRNA lentiviruses at 1 or 10 MOI and luciferase activity was measured at 2–21 days post-infection (dpi). (**c,d**) Indicated HIV-1 EGFP reporter T cell lines were infected with dCas9-VPH and indicated msgRNAs lentiviruses at 10 MOI and flow cytometric analysis for EGFP percentage (**c**) and individual intensity (**d**) was performed at 1 dpi. (**e,f**) HIV-1 latent CHME5 microglial cells were infected at 10 MOI and flow cytometry was performed at 1 dpi. All data represent relative changes (n = 3–4) to the corresponding empty msgRNA control (Zero). *p < 0.05 and **p < 0.01 indicate statistical significance by ANOVA and FLSD test.

To examine the latency-reversing efficiency of the identified msgRNAs LTR-L and O in TZM-bI HIV-1 latent cells, we established a dCas9-VPH-expressing stable cell line by lentivirus infection and double selection with puromycin for dCas9-VP64 and hygromycin for MS2-p65-HSF1 followed by lentiviral transduction with indicated msgRNAs. Such a strategy ensured a higher feasibility of expressing all three genes in a single cell and thus increased the gene delivery efficiency in TZM-bI cells. We found that msgRNA LTR-L and O induced robust activation of LTR-eLuc reporter in a dose- and time-dependent manner, while msgRNA-I, M, N showed no effect (Fig. 3a,b). At 21 days post-infection (dpi), the reactivation efficiency reached 115-fold (Fig. 3b). The effective reactivation with msgRNA LTR-L and O is consistent with the finding above in HEK293T cells using transiently transfected EcoHIV-eLuc reporter. These data suggest that the latency-reversing potency by LTR-L and O is specific, accumulative and robust.

### Preclinical application of LTR-L/O msgRNAs-induced reactivation in HIV-1 latently-infected cell lines

Memory T cells are the best-studied HIV-1 latent reservoirs. To explore the feasibility of dCas9-SAM in reactivating HIV-1 provirus in latent T cells, we performed an HIV-1 EGFP reporter assay using several HIV-1 latent T cell lines, including J-Lat^30^, 2D10, 3C9 and E4^29^,^31^. Co-transduction of all dCas9-SAM components via a lentiviral gene delivery system resulted in a dramatic increase in the number of reactivated EGFP^+^ cells (Fig. 3c) and relative fluorescence intensity per EGFP^+^ cells (Fig. 3d). Various reactivation levels in different T cell lines may result from different transduction efficiencies and perhaps transcriptional mechanisms^31^,^32^.

To evaluate the latency-reversing properties of the dCas9-SAM system in a brain latent reservoirs, we conducted similar experiments using a CHME5 microglial cell line, a well-established model to study the latent infection and reactivation in NeuroAIDS (Supplemental Fig. S1a)^33^. After cotransduction of dCas9-VPH and indicated msgRNAs, both the percentage (Fig. 3e) and the intensity (Fig. 3f) of EGFP expression were increased dramatically in LTR-L and O. A higher reactivation efficiency in CHME5 cells than T cells may be attributed to a higher efficiency of gene delivery in CHME5 cells.

### Sustained/increasing reactivation and virus-induced suicide death

**Figure 4:**
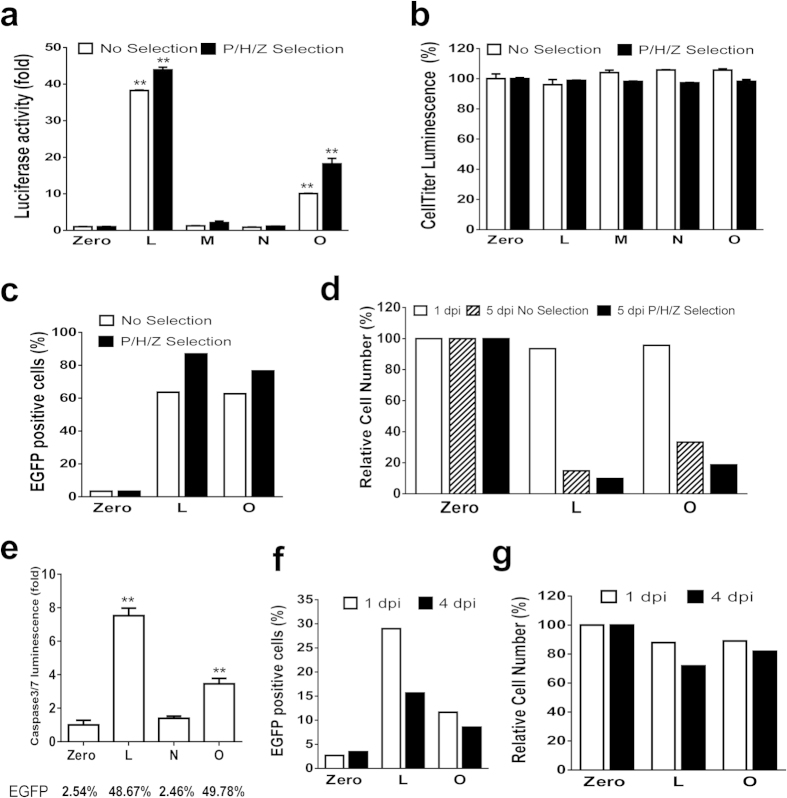
Potent and persistent reactivation of HIV-1 LTR promoter induces suicide death of HIV-1 latent 2D10 T and CHME5 microglial cells but not TZM-bI cells due to the production of toxic viral proteins. (**a**,**b**) TZM-bI cells were infected with indicated lentiviruses for 1 d and triple antibiotic selection with puromycin/hygromycin/zeoin (P/H/Z) was maintained for 4 d before passage, OneGlo luciferase reporter assay (**a**) and CellTiter-Glo® luminescent cell viability assay (**b**). (**c,d**) CHME5 cells were infected with indicated lentiviruses and cultured for 5 d in the absence or presence of P/H/Z triple selection. Flow cytometry analysis was performed for reactivation efficiency (**c**) and total survival cell number (**d**). (**e**) Caspase-Glo^®^ 3/7 Assay was performed at 2 dpi to evaluate apoptotic cell death. The reactivation efficiency was validated by flow cytometry (EGFP % on the bottom). (**f,g**) The 2D10 cells were infected for 1 and 4 d, and the reactivation efficiency (**f**) and relative total survival cell number (**g**) were determined by flow cytometry. *p < 0.05 and **p < 0.01 indicate statistical significance by ANOVA and FLSD test.

As shown in Fig. 3b, we demonstrated that a lentivirus-mediated dCas9-VPH/msgRNA system was capable of sustaining HIV-1 reactivation over the time course (21 d) of the experiments. To further pursue this, we passaged the infected cells in the presence or absence of antibiotic selection (to clear out any uninfected cells). We found that TZM-bI cells retained the growth capacity and the continuous LTR activation even after 3 passages (Fig. 4a). There was no significant difference in the cell proliferation/survival (Fig. 4b) between the activation-effective groups (LTR-L and O) and the activation-ineffective groups (LTR-Zero, M, N). Triple antibiotic selection enriched the infected cells and increased the activation efficiency as expected, but did not kill a significant number of cells (<2–7%) due to a high infection efficiency. This finding implies that the LTR-eLuc reporter system in TZM-bI cells does not produce toxic viral proteins as designed.

In contrast, we were unable to continuously passage the CHME5 and 2D10 cells due to continuous apoptosis of the latency-reversed cells via toxic viral protein buildup (Fig. 4c–g). Both CHME5 and 2D10 cell lines contain the HIV-1 genome with a deletion in the partial Gag domain and full Pol domain and an insertion of the d2EGFP encoding sequence near the 3′-LTR^31^,^33^. Both generate toxic viral proteins such as Tat, Vpu, Rev, Env, etc (Supplemental Fig. S1a). For the first passage of CHME5 cells at 5 dpi, the reactivation percentage (Fig. 4c, Supplemental S1b,c), total fluorescence intensity and individual fluorescence intensity (Supplemental Fig. S2a) for the EGFP reporter remained high in LTR-L and O groups while comparing to that at 1 dpi (Fig. 3f). Interestingly, the sustained reactivation by both LTR-L and O induced cell death in more than 80% of infected cells at 5 dpi (Fig. 4d), corresponding to the high efficiency of lentivirus infection. Similarly, triple antibiotic selection enriched the infected cells and increased the reactivation efficiency and cell death moderately as expected (Fig. 4c,d). When the LTR-Zero control cells grew to confluence for passage, the number of cells with effective reactivation by LTR-L or O was too small to be analyzed with flow cytometry. When the LTR-Zero control cells reached the third passage, the reactivated cells in LTR-L or O groups were completely lost as visualized under fluorescent microscopy. A caspase-3/7 apoptotic assay verified a significant increase in the number of apoptotic cells after LTR-L and O induced reactivation (Fig. 4e). Interestingly, LTR-O and LTR-L induced similar levels of HIV-1 provirus reactivation (Fig. 4c, Supplemental Fig. S2a), but the apoptotic cell death was significantly less in LTR-O than that in LTR-L (Fig. 4d,e), suggesting that LTR-L and O may affect different patterns of viral protein expression.

For 2D10 cells, the viral protein-induced cell death was less (10–28%) compared to CHME5 cells probably due to lower reactivation efficiency (Figs 3c and 4f,g, Supplemental Fig. S2b,c). The percentage and total fluorescence intensity of the reactivated EGFP was reduced at 4 dpi from that at 1 dpi due to viral protein-induced cell death (Fig. 4g, Supplemental Fig. S2b,c). However, the fluorescence intensity in single reactivated cells remained elevated at 4 dpi (Supplemental Fig. S2b), indicating sustained reactivation of latent HIV-1 EGFP reporter.

**Figure 5:**
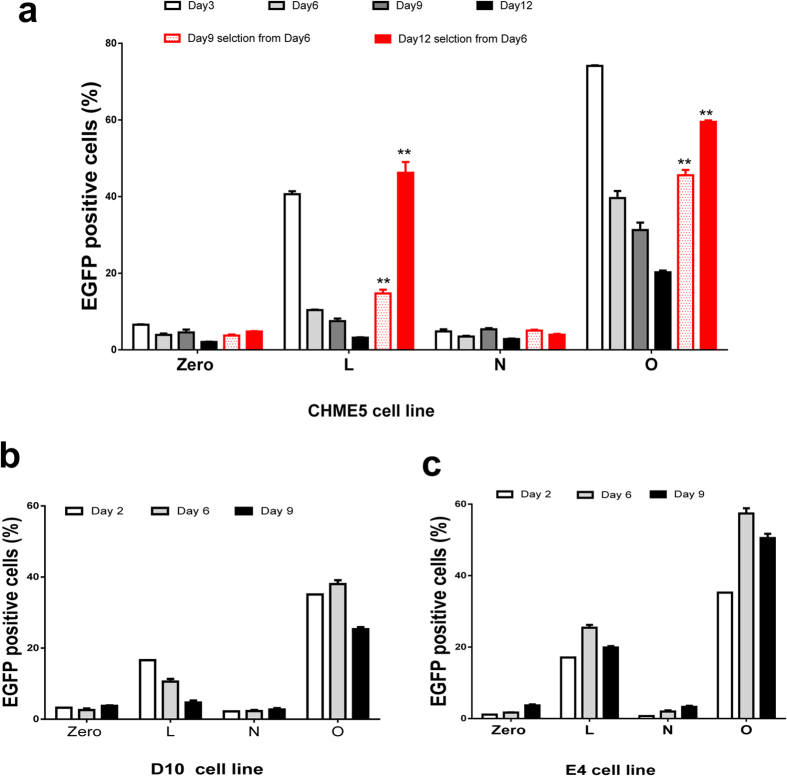
Potent and persistent reactivation of HIV-1 EGFP reporter virus and suicide cell death by dCas9-VPH/msgRNA in CHME5 microglial cells (**a**) and Jurkat-derived 2D10 (**b**) or E4 (**c**) T cell lines. (**a**) Time-dependent reduction in EGFP^+^ CHME5 cells due to continuous reactivation and suicide cell death. P/H/Z triple selection enriched lentivirus-infected cells and validated continuous EGFP reporter reactivation. **p < 0.01 indicate statistical significance by student’s *t* test as compared to corresponding non-selection groups. (**b**,**c**) Time-dependent reduction in EGFP^+^ 2D10 or E4 cells due to continuous reactivation and suicide cell death.

During continuous culture, the numbers of reactivated CHME5 (Fig. 5a) and Jurket-derived T cells (Fig. 5b,c) were gradually reduced due to cell death, which is in contrast to the sustained and increasing reactivation of LTR-luciferase reporter in TZM-bI cells (Fig. 3b). Again, the declination of reactivation was slower in LTR-O than LTR-L in CHME5, 1D10 and E4 cells (Fig. 5), implying that LTR-O msgRNA may promote the expression of some anti-apoptotic viral proteins or probably other cellular proteins in these cell lines. This was further evidenced in J-lat and 3C9 cell lines, in which the majority of cells died at the first passage after introducing LTR-L or O by lentiviral transduction. These data suggest that the provirus reactivation response and mechanisms varied with different latent cell lines, which is consistent with previous reports^31^,^32^. Selective and sustained reactivation of the latent HIV-1 reservoirs induces suicide death via the toxic viral proteins but does not affect the survival of neighboring non-reactivated cells.

### The advantage of dCas9-SAM-induced LTR reactivation over latency-reversing agents

**Figure 6:**
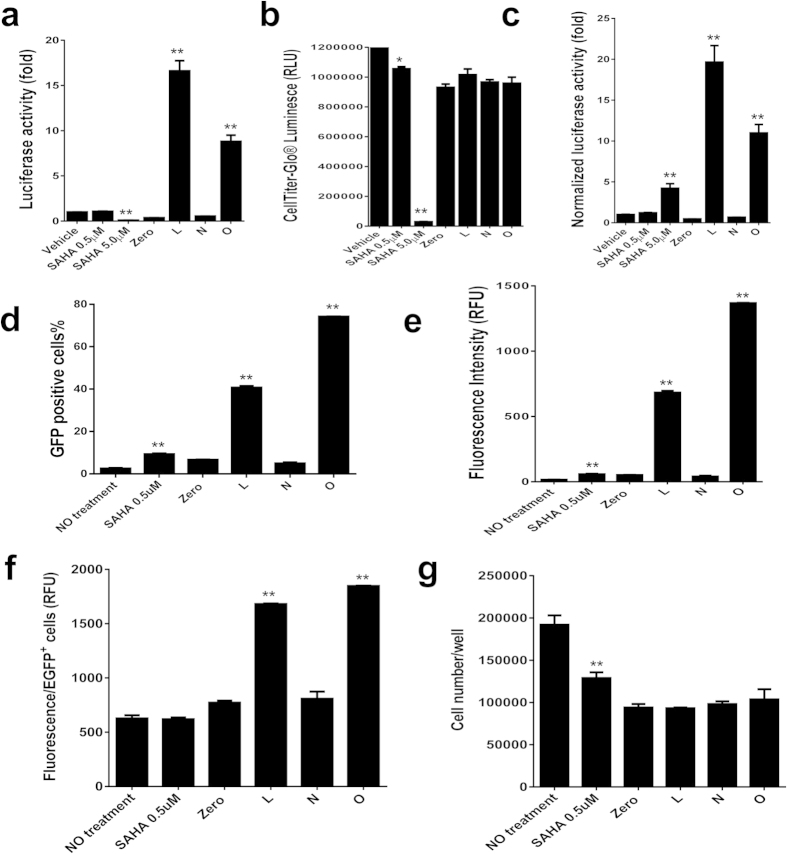
Robust reactivation of HIV-1 LTR by dCas9-SAM/msgRNAs but not SAHA in TZM-bI and CHME5 cells. (**a–c**) The dCas9-VPH stable TZM-bI cells were treated with SAHA or infected with indicated msgRNA lentivirus for 2–4 d before luminescence reporter assay (**a**) and CellTiter-Glo^®^ luminescent cell viability assay (**b**). Relative reactivation change was normalized by cell viability (**c**). (**d–g**) The dCas9-VPH stable CHME5 cells were treated with SAHA or infected with indicated msgRNA lentivirus for 4 d before EGFP flow cytometry for reactivation efficiency (**d**), total (**e**) and individual (**f**) fluorescent intensity and survival cell number (**g**). All data represent mean ± SEM of 3–4 independent experiments. *p < 0.05 and **p < 0.01 indicate statistical significance by ANOVA and FLSD test as compared with corresponding vehicle control or LTR-zero control.

Several latency-reversing agents have been identified to “shock” the HIV-1 latent reservoirs^2^,^3^,^4^,^5^. The HDAC inhibitor SAHA is widely used in clinical practice and holds a promising application in HIV-1 cure with the “shock and kill” strategy^34^,^35^. However, HDAC inhibition does not activate HIV-1 latency in some cases due to different epigenetic mechanisms^6^,^34^. To determine if the dCas9-SAM system functions better than currently used latency reversing agents in terms of cellular response, efficiency, specificity and persistence, we chose SAHA as a representative example. We selected TZM-bI cells because the integrated LTR promoter is poorly responsive to SAHA (Fig. 6a)^36^. A higher dose (500 nM) of SAHA did not activate the LTR-eLuc reporter, while a super-high dose (5,000 nM) induced 4-fold reactivation, but killed all the cells (Fig. 6b,c). In contrast, the dCas9-VPH/msgRNA LTR-L and O induced robust reactivation but did not significantly cause any cell death as compared with LTR-Zero (Fig. 6a–c). The reduced cell viability in the LTR-Zero group as compared to the vehicle control group may reflect the growth inhibition by a higher titer of lentivirus. Interestingly, continuous passaging and triple antibiotic selection produced an increasing reactivation up to 115-fold at 21 dpi in TZM-bI cells (Fig. 3b). To further evaluate a better reactivation of latent provirus by the dCas9-VPH/msgRNA, we compared the effect of SAHA in CHME5 cells. Similar to TZM-bI cells, high dose (500 nM) of SAHA induced a marginal increase in HIV-1 provirus reactivation (Fig. 6d–g). However, the dCas9-VPH/msgRNA LTR-L and O induced robust reactivation at 4 dpi in CHME5 cells(Fig. 6d–g). These data suggest that dCas9-SAM technology induces a compulsory reactivation independent of the latency mechanism, and exhibits better reactivation efficiency and specificity than SAHA.

### Additive activation of HIV-1 virus by multiplex LTR-msgRNAs

As described above, we identified 2 msgRNAs with the highest efficiency targeting −145 and −92 bp from the transcriptional start site (TSS) within the HIV-1 LTR promoter (Supplemental Table S1). We hypothesize that multiplex msgRNAs may produce synergistic and/or additive action. To test this, we coexpressed msgRNAs LTR-L and -O in dCas9-VPH expressing HEK293T cells and performed an EcoHIV-eLuc reporter assay. As shown in Supplemental Fig. S3, co-transfection of msgRNA LTR-L and -O induced additive activation of the EcoHIV-eLuc reporter. These data suggest that multiplex msgRNAs enriched transcriptional activators within the LTR promoter regions and ultimately increased the reactivation efficiency.

## Discussion

**Figure 7:**
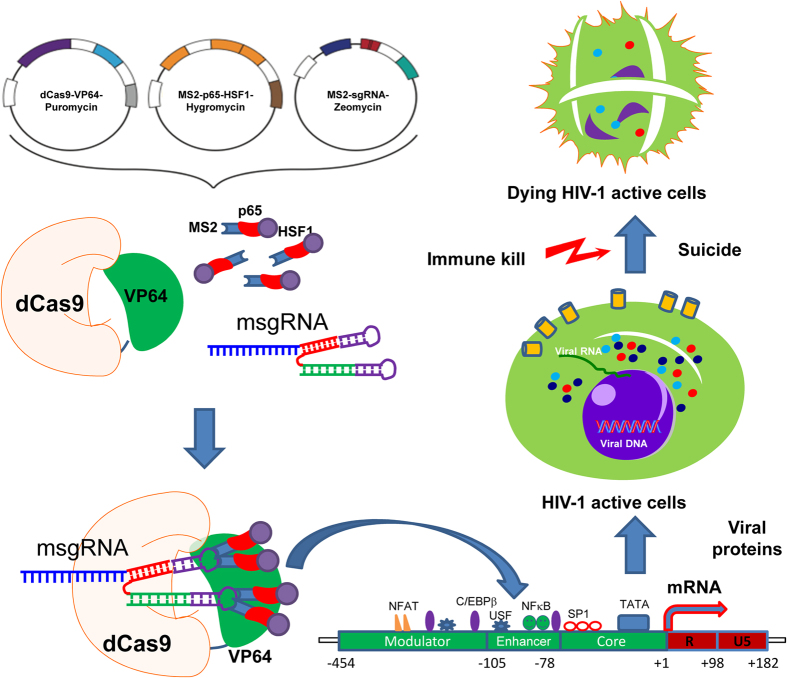
Overview of the dCas9-VPH SAM system using MS2-mediated sgRNA to direct multiple activators to the enhancer region of the HIV-1 promoter and reactivate viral protein expression in HIV-1 latent cells, which further induce suicide death and/or trigger host immune response to kill HIV-1 latent cells.

HIV-1 latent cellular reservoirs persist even in the cART era, stalling the road to a permanent cure for HIV-1 infection. As of now, complete elimination of HIV-1 latent reservoirs from the whole body remains a big challenge. Two promising strategies to cure HIV/AIDS have been developed: proviral genome eradication^29^ and latency-reversal in reservoirs cells^1^,^7^. The latter strategy, well known as “shock and kill” (also dubbed “kick and kill” or “reactivation and elimination”), is widely employed to wake up the dormant proviruses for subsequent clearance of latently-infected cells by viral cytotoxicity and/or host immune defense mechanisms^2^,^3^,^4^. In this study, we conceptually and technically demonstrated for the very first time the successful reactivation of HIV-1 latent proviruses by a novel dCas9-SAM technology. The salient finding of this study is the identification of highly effective and specific msgRNAs that induce sustained reactivation of the HIV-1 latent provirus and ultimately result in suicide death of HIV-1 infected cells (Fig. 7).

Our HIV-1 latency-reversing system is innovative and exhibits several advantages over the current “shock and kill” strategy with latency-reversing chemicals or agents for the following reasons: (1) *High Specificity*: Target-specific msgRNAs were carefully designed through bioinformatics analysis and guided the novel dCas9-SAM to the HIV-1 promoter exclusively in HIV-infected cells. Additionally, we identified a very short region (around the enhancer adjacent to NF
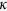
B binding sites) that is responsive to the dCas9-VPH reactivation. Finally, the suicide cell death is specifically dependent upon the production of HIV-1 toxic viral proteins. (2) *High Efficiency*: This dCas9-SAM technology delivers multiple exogenous transcriptional activators to the target site(s) and induces substantial increases in target cellular gene expression as compared to a single activator system^11^,^27^,^28^. This study verified that the dCas9-SAM is more efficient than dCas9-VP64 alone in activating proviral production. This activation efficiency would not be affected by HIV-1 integration sites and endogenous epigenetic modification, which is a major obstacle for HIV-1 latency reactivation by the currently used chemicals or agents. (3) *Sustained Reactivation Until All HIV-1 Latent Cells are Killed*: The dCas9-VPH and msgRNAs are continuously expressed and/or inducibly controlled. Importantly, the dCas9 does not induce any indel mutation of the msgRNA target site that may prevent further binding of msgRNA to the targets (no self-limit). Therefore, the dCas9-SAM system is capable of retaining persistent levels of compulsory reactivation, leading to sustained and unlimited generation of viral proteins that will consequently kill the HIV-1 latent cells. This feature is very important for HIV-1 “shock and kill” strategy. (4) *No*/*low Cytotoxicity to Neighboring HIV-Negative Cells*: The dCas9 enzyme does not contain any nuclease activity, and thus would not induce any mutation or chromosome translocations/instabilities in the host cells. The dCas9-SAM system only affects HIV-infected cells, in contrast to currently developed chemical agents that exhibit non-specific effects on other non-infected cells. Even though the HIV-1 msgRNAs may have potential off-target sites (particularly with mismatch) in the host genome (extremely rare as shown in Cas9-sgRNA system), the possibility of the recruited dCas9-SAM complex to activate any potential pathogenic genes is extremely low because only 1.2% of genome encode functional genes and the number of pathogenic genes is extremely limited. (5) *More msgRNAs Can Increase Proviral Reactivation Efficiency*: As demonstrated in this work, multible msgRNAs can be developed to increase reactivation efficiency. The synergistic action of LTR-L and LTR-O provides us a guide to develop an all-in-one viral or non-viral gene delivery system for further preclinical (animal) and clinical (patient) studies.

The ultimate goal of the latency-reversing strategy is to eliminate (kill) HIV-1-infected cells through toxic viral protein buildup and/or host immune clearance. In the culture system, the viral proteins play a major role in killing HIV-1 latent cells. In this study, the cell killing effect induced by the dCas9-VPH/msgRNAs system is completely dependent upon the generation of viral proteins as evidenced by (1) LTR potent reactivation induced cell death only in those cell lines that harbor the HIV-1 proviral genome (CHME5, 2D10, etc.) but not in TZM-bI cells that contain only the LTR-luciferase reporter; (2) Cell death depends upon the extent of LTR reactivation; (3) Establishing a stable cell line carrying the HIV-1 provirus and dCas9-VPH/msgRNAs was impossible or difficult due to viral protein-induced cell death. The identification of the specific viral proteins that induce suicide cell death during dCas9-SAM reactivation warrants further investigation.

By screening different sgRNAs or msgRNAs using dCas9-single activator or multiple activators, we found that the activation efficiency varied with different target sites as well as different dCas9 systems. This study focuses on the more effective dCas9-SAM system and demonstrated that LTR-L and O are the best msgRNAs to activate the HIV-1 LTR promoter, even though LTR-L and O exhibited various efficiencies of reactivation and apoptotic induction in different cell lines. However, it is likely that other combinations of multiple transcriptional activators^27^,^28^ may present different sgRNAs with best efficiency. It is also likely that LTR-L and O regulate various viral proteins in a different expression manner and through different molecular mechanisms.

Potential off-target effects remain a critical concern for the preclinical and clinical application of the CRISPR/Cas9 and its derived dCas9 system. Several promising strategies have been developed to mitigate any potential off-target responses, such as the sgRNA design bioinformatic optimization, transcriptome analysis, and functional screening after dCas9 treatment. For the parent Cas9-sgRNA system, more and more experimental data support that the genome editing is highly specific. Several reports using whole genome sequencing (WGS) at 15–100x coverage have demonstrated very rare instances of off-target effects while employing the Cas9/gRNA technology *in vitro*^29^,^37^,^38^,^39^,^40^. Newly developed unbiased profiling techniques further validate the high specificity of this Cas9-sgRNA system^41^,^42^,^43^. *In vivo* off-target effects are expected to be much lower due to epigenetic protection. In addition, the off-target frequency in essential gene/genome will be very rare because exons comprise only 1.2% of the entire genome. In the case of dCas9 system, the frequency of off-target binding to essential (functional) exons would also be very low. RNA-seq analysis confirmed the specificity of this dCas9-SAM technology^11^. In our study, the exogenous viral DNA was analyzed against the host genome for best score of efficiency and specificity. In TZM-bI cells without viral protein production, the dCas9-VPH/msgRNAs induced potent reactivation of LTR-eLuc reporter, but did not influence the cell growth/proliferation, supporting the absence of off-target effects by the dCas9-VPH/LTR-msgRNA system. Nevertheless, further analysis by RNA-sequencing, RT-PCR array or microarray is warranted.

The HIV-1 genome contains almost identical 5′- and 3′-end LTRs. The 5′-LTR normally functions as an RNA polymerase II promoter but the 3′-LTR acts in transcription termination/polyadenylation and is not normally functional as a promoter due to transcriptional interference^44^. Such transcriptional suppression is attributed to the competition of endogenous transcriptional factors between 5′- and 3′-LTR promoter^44^,^45^. In this study, the msgRNAs designed for 5′-LTR may also affect the 3′-LTR promoter activity because the recruitment of SAM to target specific region is independent from the endogenous transcriptional factors. Additional activation of the integrated HIV-1 provirus via 3′-LTR promoter by dCas9-SAM system might be another advantage of this novel approach over the currently-used chemical agents. This proof of concept is worthwhile of further investigation using an indicator gene downstream of the 3′-LTR^46^.

In conclusion, we have demonstrated that the latent HIV-1 provirus can be reactivated dramatically by the engineered dCas9-SAM guided by msgRNAs specifically targeting the enhancer of the HIV-1 LTR promoter (Fig. 7). This study presents a novel, early-stage preclinical approach that will result in the development of an additional “shock and kill” therapeutic strategy to eliminate the HIV-1 latent reservoirs in a clinical setting. One major factor that is slowing the dCas9-SAM system’s path to clinical trials is the low gene delivery efficiency to HIV-1 latent cells. Improvement in the viral and non-viral gene delivery in the field of gene therapeutics will boost the clinical application of the CRISPR/Cas9 technology.

## Methods

### Plasmids and cloning of sgRNA or msgRNA expression and EcoHIV-eLuc vectors

The plasmids used are gifts from indicated citations: pMSCV-LTR-dCas9-VP64-BFP (Addgene, plasmid #46912)^21^, pHAGE TRE dCas9-VP64(Addgene plasmid #50916)^47^, and lenti-MS2-P65-HSF1-Hygro (Addgene, plasmid #61426)^11^.

The lentiviral vector pLV-EF1α-dCas9-VP64-BFP was generated by cloning the *Bgl*II/*Xho*I-digested fragment of dCas9-VP64-BFP from retroviral vector pMSCV-LTR-dCas9-VP64-BFP (Addgene plasmid #46913)^21^ into lentiviral vector pLV-EF1α-Cas9-T2A-RFP via *Bam*HI/*Sal*I (Biosettia Inc). The pNL4-3-EcoHIV-eLuc vector was generated by infusion PCR^48^ with indicated primers (Supplemental Table S2). The eLuc gene, a P2A self-cleaving peptide^49^, and N-terminal of HIV-1 Nef in frame with HIV-1 splicing acceptor for HIV-1 Nef expression were amplified and then cloned into the *Bam*HI and *Xho*l restriction sites of the HIV-1 proviral clone pNL4-3 (from Dr. Malcolm Martin through the NIH AIDS Reagent Program, Division of AIDS, NIAID, NIH)^50^. To generate pNL4-3-EcoHIV vector (Supplemental Fig. S3), the coding region of gp120 in HIV-1 pNL4-3 was replaced with gp80 from ecotropic murine leukemia virus (MLV), which was PCR-amplified from pHCMV-EcoEnv (Addgene plasmid 15802, a gift from Dr. Miguel Sena-Esteves)^51^ following the engineering strategy previously published^52^.

The seed sequences targeting the HIV-1 LTR (634 bp) were predicted by using the Broad Institute sgRNA designer tool for highly effective sgRNA design and MIT’s CRISPR Design for the off-target prediction (http://CRISPR.mit.edu). Both sense and antisense target sequences using NGG as the PAM were described previously^29^, from which 22 target sites with high score of cleaving efficiency and specificity against human genome were selected. A pair of oligonucleotides for each targeting site with 5′-CACC and 3′-AAAC overhang was obtained from AlphaDNA (Supplemental Table S1). For sgRNAs in the dCas9-single activator system, the target seed was cloned via modified *Bbs*I sites into pKLV-WG lentiviral vector derived from pKLV-gRNA(*Bbs*I)-Puro-2A-BFP lentiviral vector (Addgene #50946)^53^. For msgRNAs in the dCas9-SAM system, the seed sequence was cloned via *Bsm*BI sites into Lenti sgRNA(MS2)-zeo backbone (Addgene, Plasmid #61427)^11^. The vectors were digested with *Bbs*I or *Bsm*BI, treated with Antarctic Phosphatase, and purified with a Quick nucleotide removal kit (Qiagen). Equal amount of complementary oligonucleotide was mixed in T4 polynucleotide kinase (PNK) buffer for annealing. These annealed seed pairs were phosphorylated with T4 PNK and ligated into the *Bbs*I or *Bsm*BI-digested lentiviral vector using T4 ligase. The ligation mixture was transformed into Stabl3 competent cells. Positive clones were identified by PCR screening and verified by Sanger sequencing using Flap or U6 primer (Supplemental Table S2).

### Cell culture

TZM-bI reporter cell line from Dr. John C. Kappes, Dr Xiaoyun Wu and Tranzyme Inc^26^, and J-Lat full length clone from Dr. Eric Verdin^30^ were obtained through the NIH AIDS Reagent Program, Division of AIDS, NIAID, NIH. CHME5/HIV fetal microglial cell line and Jurkat-derived T cell line 2D10, 3C9, E4 were donated by Dr. Jonathan Karn^31^,^32^,^33^. TZM-bI and CHME5 cells were cultured in Dulbecco’s minimal essential medium high glucose supplemented with 10% heat-inactivated fetal bovine serum (FBS) and 1% penicillin/streptomycin. J-Lat, 2D10, 3C9, and E4 T cells were cultured in RPMI1640 containing 2.0 mM L-glutamine, 10% FBS and 1% penicillin/streptomycin.

### Lentivirus and retrovirus packaging and infection

All recombinant lentiviruses or retroviruses were produced after calcium phosphate-mediated transient transfection of related vectors according to standard protocols. Briefly, HEK293T cells were cotransfected with the lentiviral transfer vector (10 μg for gRNA, 15 μg for others), lentiviral packaging vectors pRSV-REV (3 μg) and pMDLg/pRRE (8 μg), and vesicular stomatitis virus G glycoprotein (VSVG) expression vector pMD2G (5 μg). For retrovirus, the GP2-293 cells were cotransfected with retroviral vector pMSCV-LTR-dCas9-VP64-BFP (15 μg) and pMD2G (15 μg). The viruses were collected from the culture supernatant on days 2 and 3 post-transfection, concentrated by ultracentrifugation for 2 h at 25,000 rpm, and then resuspended in phosphate-buffered saline (PBS). Virus titer determination was performed by infecting HEK293T cells with serial diluted lentiviruses and counting the number of fluorescent protein-expressing cells 48 h post-infection under fluorescent microscopy. For a typical preparation, the titer was approximately 4−10 × 10^8^ IU/ml for gRNA and 4−10 × 10^7^ IU/ml for others. The experimental cells were infected at 10 MOI of indicated lentivirus in the presence of polybrene (8 μg/ml) by centrifugation at room temperature at 400 g for 2 h. After infection, cells were cultured for next experiments or drug selection.

### Stable cell lines

TZM-bl, CHME5 or HEK293T cells were seeded in 24-well plates at 2 × 10^4^ cells/well and transduced at 10 MOI with pMSCV-dCas9-BFP (Puromycin) and Lenti-MS2-p65-HSF1 (hygromycin). After 2 days, cells were subcultured in 6-well-plate and selected with puromycin (2 μg/ml) and hygromycin (200 μg/ml). After two weeks of selection culture, cells were seeded in 24-well plates at 2 × 10^4^ cells/well, and infected with indicated msgRNA lentivirus (10 MOI). After 2 days, cells were subcultured in 6-well-plate and selected with triple antibiotics: puromycin (0.5 μg/ml), Hygromycin (100 μg/ml) and Zeomycin (100 μg/ml).

### *Firefly*-luciferase reporter assay

Cells were cultured in a 96-well plate and transfected or transduced with indicated vectors. To examine the eLuc reporter activity, the cell lysate was prepared using the ONE-Glo luciferase assay system (Promega) and luminescence was measured in a 2104 EnVision® Multilabel Reader (PerkinElmer). Representative results were presented as mean ± SEM of 4–6 independent samples.

### EGFP flow cytometry

Cells were trypsinized (for CHME5) or collected (for suspension T cell lines), washed with PBS and fixed in 2% paraformaldehyde for 10 min at room temperature. Then, cells were washed twice with PBS and analyzed using a Guava EasyCyte Mini flow cytometer (Guava Technologies).

### Cell growth/proliferation assay

The cell growth/proliferation was determined by the trypan blue exclusion hemocytometry, and CellTiter-Glo luminescence viability assay (Promega). The CellTiter-Glo luminescent cell viability assay is a homogeneous and sensitive method to quantitate ATP generated by metabolically active cells that associates with the number of viable cells. Briefly, cells were cultured in sterile 96-well plates for indicated period and treated with 100 μl of CellTiter-Glo reagent for 10 min at room temperature. The luminescence in each well was measured in a 2104 EnVision® Multilabel Reader (PerkinElmer).

### Cell apoptosis assay

The dCas9-VPH stably-expressing CHME5 cells were seeded in 96-well-plate (2,000 cells/well) and infected with indicated msgRNA lentiviruses. At 2 dpi, the caspase-3/7 activities were examined using a Caspase-Glo^®^ 3/7 luminescence assay kit (Promega, Madison, WI) according to the product manual.

### Statistical analysis

The quantitative data represented mean ± standard error from 3–5 independent experiments, and were evaluated by ANOVA and Fisher’s LSD multiple comparison test, or unpaired Student’s *t*-test in some cases. A statistically significant difference was marked as *and **for p value < 0.05 and 0.01 respectively.

## Additional Information

**How to cite this article**: Zhang, Y. *et al.* CRISPR/gRNA-directed synergistic activation mediator (SAM) induces specific, persistent and robust reactivation of the HIV-1 latent reservoirs. *Sci. Rep.* **5**, 16277; doi: 10.1038/srep16277 (2015).

## Supplementary Material

Supplementary Information
