# Supplementary material for: CRISPR/gRNA-directed synergistic activation mediator (SAM) induces specific, persistent and robust reactivation of the HIV-1 latent reservoirs
Source: Sci Rep. 2015 Nov 5;5:16277. doi: 10.1038/srep16277 (PMC4633726; doi:10.1038/srep16277)
Supplement: Supplementary Information [file srep16277-s1.pdf]

**Supplemental Information**

# CRISPR/gRNA-directed synergistic activation mediator (SAM) induces specific, persistent and robust reactivation of the HIV-1 latent reservoirs

Yonggang Zhang[1], Chaoran Yin[1], Ting Zhang[1], Fang Li[1], Wensheng Yang[1], Rafal Kaminski[1], Philip Regis Fagan[1], Raj Putatunda[1], Won-Bin Young[2], Kamel Khalili[1*] and Wenhui Hu[1*]

[1]Department of Neuroscience, Center for Neurovirology and The Comprehensive NeuroAIDS Center, Temple University School of Medicine, 3500 N Broad Street, Philadelphia, PA 19140; [2]Department of Radiology, University of Pittsburgh School of Medicine, Pittsburgh, PA, 15219

* Corresponding authors:

Wenhui Hu, MD, PhD, Department of Neuroscience and Center for Neurovirology, Temple University School of Medicine, 3500 N Broad Street, Philadelphia, PA 19140, USA; Tel: 1-215-707-5164, Fax: 1-215-707-4888, E-mail: whu@temple.edu

Kamel Khalili, Ph.D. Department of Neuroscience and Center for Neurovirology, Temple University School of Medicine, 3500 N. Broad Street, Philadelphia, PA 19140; Tel: 215-707-4500, Fax: 215-707-4888, Email: kamel.khalili@temple.edu

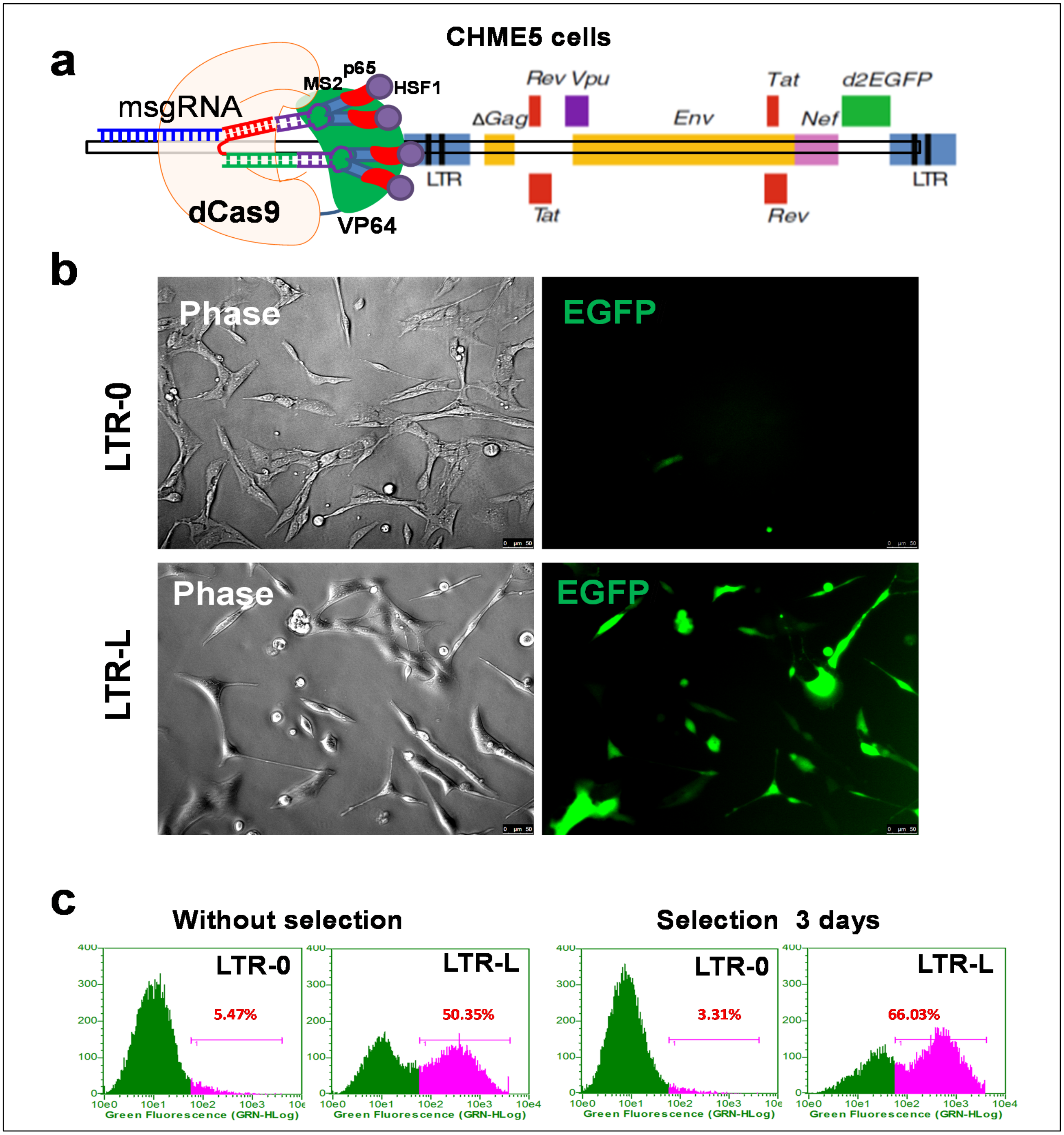


**Fig. S1. Robust and persistent reactivation of HIV-1 EGFP reporter by dCas9-VPH/msgRNA in latent CHME5 microglial cells. (a)** Illustration of dCas9-VP64/MS2-p65-HSF1 (VPH) and MS2-mediated single guide RNAs (msgRNAs) in activating HIV-1-EGFP reporter virus in CHME5 cells. (**b, c**) Cells were infected with pMSCV-LTR-dCas9-VP64-puromycin and Lenti-MS2-p65-HSF1-hygromycin (dCas9-VPH) and selected with puromycin (1 μg/ml) and hygromycin (1 μg/ml) for 10 days. The population cells were infected with indicated msgRNA-Zeocin lentivirus. The fluorescent micrographs were taken at 2 d postinfection (**b**). Cells were continuously cultured and selected with triple antibiotics (puromycin-hygromycin-Zeocin) for 3 d before flow cytometry with EGFP (**c**).

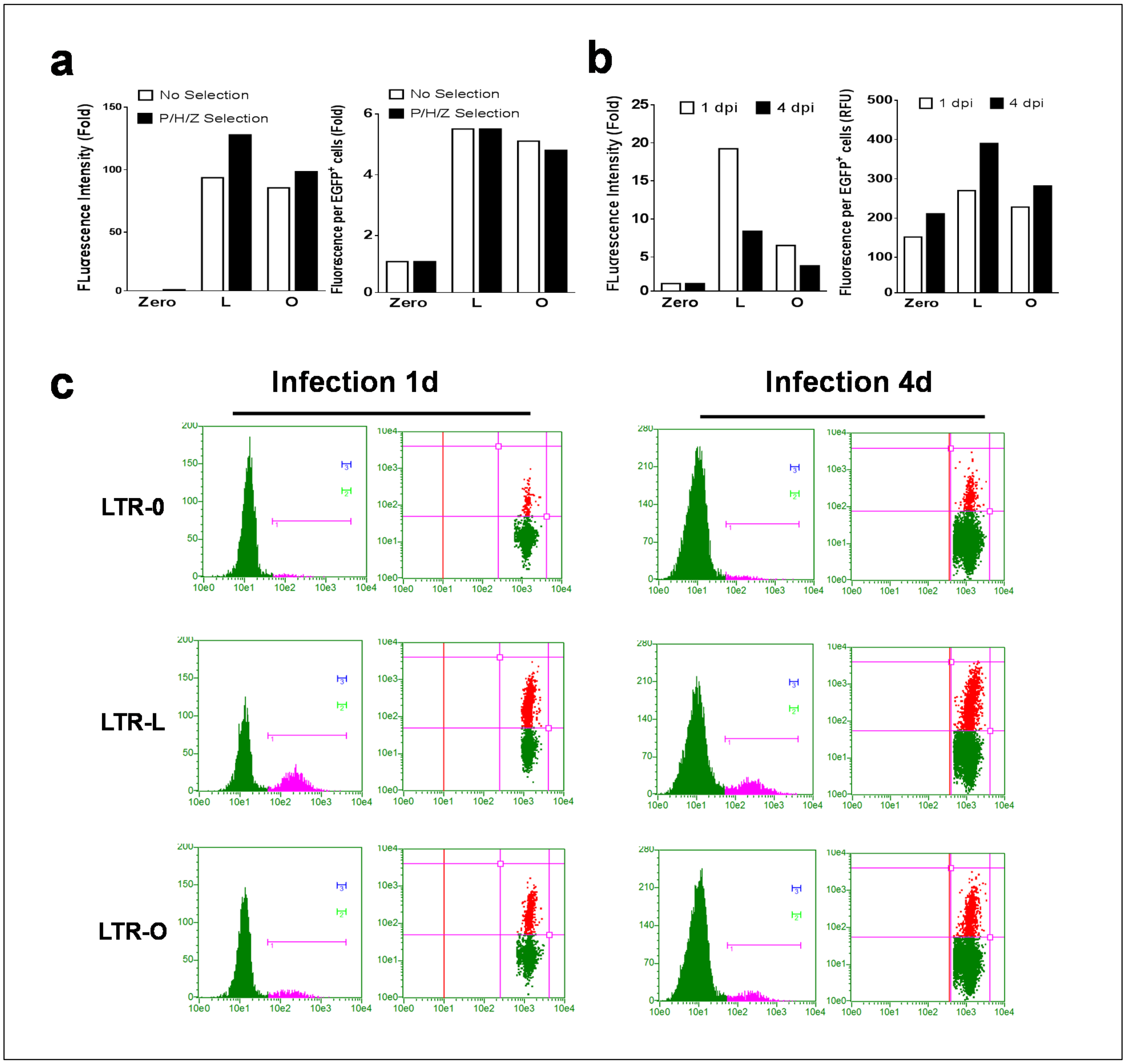


**Fig. S2. Persistent reactivation of HIV-1 EGFP reporter virus and suicide cell death by dCas9-VPH/msgRNA in HIV-1 latent cells.** (**a**) CHME5 microglial cells were infected with indicated lentiviruses and cultured for 5 d in the absence or presence of P/H/Z triple selection (puromycin-hygromycin-Zeocin). Flow cytometry analysis was performed for total and individual EGFP fluorescent intensity. (**b, c**) The 2D10 Jurkat T cells were infected for 1 and 4 d, and flow cytometry was performed for total and individual EGFP fluorescent intensity (**b**) and the EGFP reactivation efficiency (**c**).

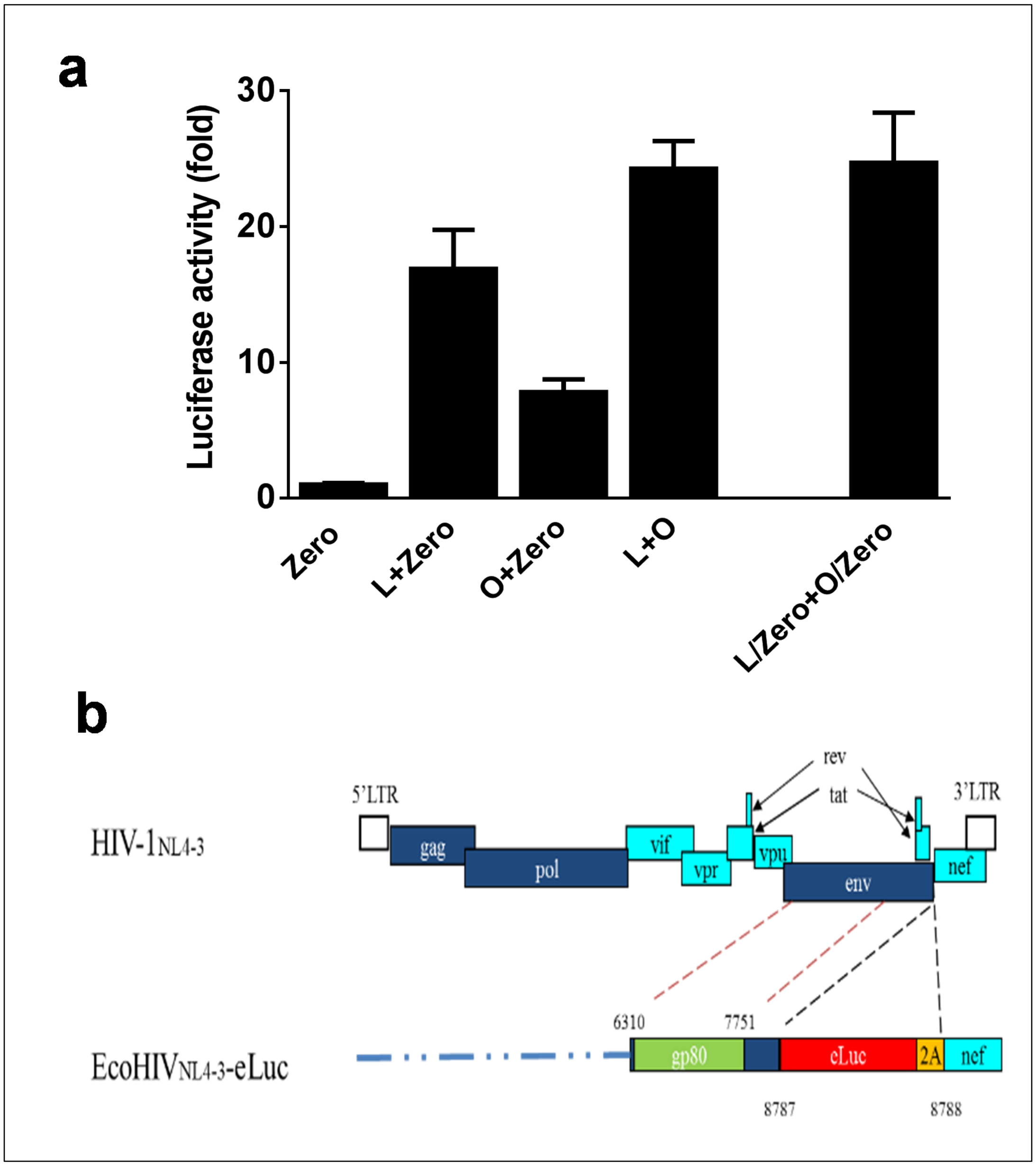


**Fig. S3. (a) LTR-L and O induced additive reactivation of EcoHIV-eLuc reporter.** The dCas9-VPH HEK293T cells were transfected with an equal amount of indicated msgRNAs LTR-L or O followed by ONE-Glo™ luciferase assay. Data represent mean ± SEM of 5 independent transfections. The right column shows the additive data from L+Zero and O+Zero groups. **(b) Schematic representation of EcoHIV-1 containing enhanced *firefly* luciferase (eLuc).** The eLuc gene was inserted after env which is driven by the 5'-LTR after the alternative splicing. For bicistronic expression, a self-cleaving 2A peptide from porcine teschovirus-1(P2A) was inserted between eLuc and nef. The envelope of HIV-1 was replaced with gp80 from murine leukemia viruses for construction of EcoHIV.

## Table S1. Sequences and locations for msgRNA targeting sites

| Target name | To TSS | Direction | Sequences (5' to 3') |
|---|---|---|---|
| LTR-C | -357 to -377 | T357: Sense | caccGATTGGCAGAACTACACACC |
| | | T358: Antisense | aaacGGTGTGTAGTTCTGCCAATC |
| LTR-D | -55 to -75 | T359: Sense | caccGCGTGGCCTGGGCGGGACTG |
| | | T360: Antisense | aaacCAGTCCCGCCCAGGCCACGC |
| LTR-E | -390 to -412 | T361: Sense | caccGATCTGTGGATCTACCACACACA |
| | | T362: Antisense | aaacTGTGTGTGGTAGATCCACAGATC |
| LTR-F | -18 to -40 | T363: Sense | caccGCTGCTTATATGCAGCATCTGAG |
| | | T364: Antisense | aaacCTCAGATGCTGCATATAAGCAGC |
| LTR-G | -393 to -413 | T530: Sense | caccGTGTGGTAGATCCACAGATCA |
| | | T531: Antisense | aaacTGATCTGTGGATCTACCACAC |
| LTR-H | -375 to -395 | T532: Sense | caccGCAGGGAAGTAGCCTTGTGTG |
| | | T533: Antisense | aaacCACACAAGGCTACTTCCCTGC |
| LTR-I | -327 to -427 | T534: Sense | caccGATCAGATATCCACTGACCTT |
| | | T535: Antisense | aaacAAGGTCAGTGGATATCTGATC |
| LTR-J | -194 to -214 | T536: Sense | caccGCACACTAATACTTCTCCCTC |
| | | T537: Antisense | aaacGAGGGAGAAGTATTAGTGTGC |
| LTR-K | -163 to -183 | T538: Sense | caccGCCTCCTAGCATTTCGTCACA |
| | | T539: Antisense | aaacTGTGACGAAATGCTAGGAGGC |
| LTR-L | -145 to -165 | T540: Sense | caccGCATGGCCCGAGAGCTGCATC |
| | | T541: Antisense | aaacGATGCAGCTCTCGGGCCATGC |
| LTR-M | -122 to -142 | T542: Sense | caccGCAGCAGTCTTTGTAGTACTC |
| | | T543: Antisense | aaacGAGTACTACAAAGACTGCTGC |
| LTR-N | -106 to -126 | T544: Sense | caccGCTGACATCGAGCTTTCTACA |
| | | T545: Antisense | aaacTGTAGAAAGCTCGATGTCAGC |
| LTR-O | -92 to -112 | T546: Sense | caccGTCTACAAGGGACTTTCCGCT |
| | | T547: Antisense | aaacAGCGGAAAGTCCCTTGTAGAC |
| LTR-P | -81 to -102 | T548: Sense | caccGCTTTCCGCTGGGGACTTTCC |
| | | T549: Antisense | aaacGGAAAGTCCCCAGCGGAAAGC |
| LTR-Ra | 61 to 81 | T422: Sense | caccGCTTTATTGAGGCTTAAGCAG |
| | | T423: Antisense | aaacCTGCTTAAGCCTCAATAAAGC |
| LTR-U5a | 109 to 128 | T424: Sense | caccGCCCGTCTGTTGTGTGACTC |
| | | T425: Antisense | aaacGAGTCACACAACAGACGGGC |

# Table S2. Primers used for sequencing and PCR

| | |
|---|---|
| **Sequencing** | |
| hU6-sequence | ATGGACTATCATATGCTTACCG |
| Flap-Seq/5'/F | CAGTGCAGGGGAAAGAATAGTAGAC |
| mSCV-Rev | CAGCGGGGCTGCTAAAGCGCATGC |
| **PCR primer** | |
| HIV-EcoEnv-F1 | TGGGATATTGATGATCTGTAGTGCTATGGCGCGTTCAACGCTCT |
| HIV-EcoEnv-R1 | AGAGCGTTGAACGCGCCATAGCACTACAGATCATCAATATCCCA |
| HIV-EcoEnv-F2 | GCCTATAGAGTACGAGCCATAGGTGGGAATAGGAGCTTTGTTCC |
| HIV-EcoEnv-R2 | GGAACAAAGCTCCTATTCCCACCTATGGCTCGTACTCTATAGGC |
| HIV -Evn-opti-luc-F | GCTTGGAAAGGATTTTGCTATAAGCTAGCCACCATGGAAGATGCCAAGAACATCAAGA |
| HIV-Evn-opti-luc-R | TCTTGATGTTCTTGGCATCTTCCATGGTGGCTAGCTTATAGCAAAATCCTTTCCAAGC |
| Opti-luc-P2A-F | GCCAAGAAGGGCGGCAAGCCTAGGCCTGCCACGAACTTCTCTCTGTTAAAG |
| Opti-luc-P2A-R | CTTTAACAGAGAGAAGTTCGTGGCAGGCCTAGGCTTGCCGCCCTTCTTGGC |
| Opti-luc-P2A-R | CTTTAACAGAGAGAAGTTCGTGGCAGGCCTAGGCTTGCCGCCCTTCTTGGC |
